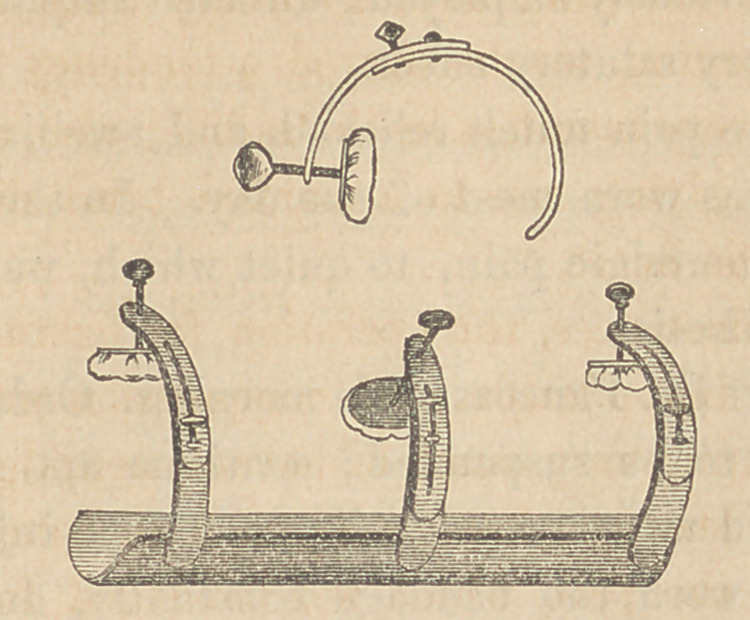# Case of Popliteal Aneurism Treated by Compression

**Published:** 1857-03

**Authors:** T. P. Gibbons

**Affiliations:** Philadelphia


ART. III.—*Case of Popliteal Aneurism treated by Compression.* By T. P. GIBBONS, M.D., of Philadelphia.

THE treatment of aneurism by compression, having recently attracted much attention both in Europe and in this country, I propose to lay before the profession the details of a particularly aggravated case that occurred in this city three years ago. I am inclined to the opinion that the subject is deserving of more attention than it has received from surgeons generally; and should the results of the operation, upon further trial, prove it to be only occasionally successful, but much less hazardous than that of ligature of the artery, a great desideratum will be supplied.

On the 23d of February, 1853, I was requested to call upon H—— B——, of this city, whom I found confined to his bed, complaining of pain and swelling of the knee, which he supposed to be caused by an attack of acute rheumatism, from which he had frequently suffered. Upon examining the part, I discovered a pulsating tumor, the size of an orange, occupying the popliteal space. The leg and foot were œdematous to a considerable degree, and the patient was unable to move the limb without assistance.

From the patient I learned the following history: He had been engaged for some years as a hotel-keeper in California, from whence he had removed to the Isthmus of Panama, to continue the same business. After a few months' residence at the latter place, during all of which time he was addicted to the excessive use of alcoholic stimuli, he contracted "Chagres Fever," from which, after a protracted illness, he partially recovered; but being disabled in finances and incapacitated for business, he concluded to return to Philadelphia, his original home. Shortly after his arrival, he determined to abandon the entire use of all stimulating liquors, a determination to which, he says, he has rigidly adhered.

Some two weeks ago, whilst engaged in closing the doors of a vault, which had become swollen by the rain, by jumping upon them, he felt "something give way in the knee." The pain which followed was slight, and gave him no great uneasiness until some days afterwards, when the knee began to swell and became very sensitive. But supposing the trouble to be owing to rheumatism, he applied the remedies which he usually resorted to in such cases, but without effect. The pain and swelling continuing to increase, he has been compelled for the past few days to keep his room.

Being satisfied of the existence of aneurism, I requested a consultation, which was had the following day, February 24th, with Dr. Pancoast. Upon careful examination, we discovered a tendency to ossification on the part of the arteries, very manifest in the radial; also, an obstruction at the mouth of the aorta, giving rise to a marked bruit de souffle, and consequent dilatation of the heart.

Under these circumstances, the operation for ligature of the femoral artery was considered inapplicable, and compression was determined upon. The patient complaining very much of severe neuralgic pain in the limb, a lotion of extract of lead and laudanum, each an ounce, to a pint of water, was applied warm to the part, which was bandaged from the toes upward to the middle of the thigh, and an anodyne mixture administered.

A proper instrument for compressing the artery not being at hand, some days elapsed before it could be procured, during which time no visible augmentation was manifest in the tumor.

March 1st.—Applied compression to the femoral artery at the inferior angle of Scarpa's triangle, by means of Skey's tourniquet, with instructions to the attendant to change the pressure to different points, according as it could be best borne. This was continued at intervals of two or three hours at a time, for twenty-six hours, when the tumefaction of the limb was so great that it was thought advisable to remove the instrument altogether. The pain, which had been very severe during the application of the instrument, became excruciating after its removal. Morphia was freely administered until four grains were taken in twelve hours, without producing the slightest tendency to drowsiness, and but partial relief of the neuralgic symptoms.

On visiting him on the evening of the 3d, I was informed that he had been delirious during the afternoon, and evidences of delirium tremens were then quite apparent. I directed an ounce of brandy to be given every hour, which, after three or four repetitions, had the effect of quieting him, when he enjoyed a few hours of broken sleep. Finding that the brandy gave more relief than anything previously tried, he resorted to the very free use of it, taking about *five pints* in forty-eight hours, during which time his condition would not admit of a reapplication of the pressure. The unsatisfactory operation of the tourniquet led me to devise the instrument here represented, which was manufactured by J. H. Gemrig, cutler, of this city.

A piece of sheet iron, curved to fit the posterior surface of the thigh, and well cushioned, forms the base of the instrument. To this, three clamp tourniquets are attached by their inferior extremities. The shaft of each tourniquet is provided with a slide, so that it can be lengthened or shortened at pleasure, and fixed at any point by means of a thumb-screw. When the instrument is in place, the pads of the three tourniquets rest immediately over the femoral artery, and by turning the screw of any one of them, the vessel is compressed against the femur. But one tourniquet is intended to be used at a time, and when the pressure exerted by this becomes too painful to be borne, either one of the others may be screwed down before relaxing the one last employed. The tourniquet pads are purposely made small, in order to avoid pressure upon the femoral vein, if possible.

March 6th.—Was summoned at 1 o'clock, A.M., and found patient delirious; directed the use of brandy to be suspended, and to give an enema of forty drops of wine of opium in an ounce of starch water, to be repeated in two hours, in case he did not sleep.

9 A.M.—Found patient somewhat relieved, but still slightly incoherent; had slept some since the repetition of the enema; directed half an ounce of liq. ammon. acet., with five grains of carb. ammon., every two hours, and at each intermediate hour eight ounces of porter; œdema of the limb slightly diminished.

1 P.M.—Consultation with Dr. Pancoast; patient somewhat easier; continue prescription; applied bandage to the limb, and adjusted the new instrument.

10 P.M.—Complains greatly of pain; compelled to remove the pressure and to bandage the limb, which is much swollen, measuring twenty-one inches in circumference at the site of the tumor; applied locally spt. camphor with chloroform.

March 7th.—Slept some last night after enema of one drachm wine of opium; swelling still continues, precluding the application of the instrument; persevere with the ammonia mixture and porter.

March 8th.—Complains this morning of his mouth feeling dry and parched. Bowels not having been moved for some days, a stimulating enema was given, after the operation of which he was much relieved. The swelling having greatly subsided, the instrument was reapplied, which he bore tolerably well for eight hours. On its removal, the swelling returned; but not to so great an extent as formerly. Pain very severe, and not yielding to the means previously employed; directed sulphuric ether, by inhalation, which had a very salutary effect.

March 10th.—The pain much relieved, and swelling abated. Applied instrument, which was worn most of the day. In the evening, had a violent attack of the neuralgic pain, to quiet which, was compelled to produce complete anæsthesia.

March 11th.—Met Dr. Pancoast this morning. Ordered the use of carb. ammonia and porter to be suspended; continue spt. mindereri, and take a glass of brandy and water occasionally; anodyne injection at night.

March 14.—Has worn the bandage constantly, and had the pressure applied from four to eight hours a day, since the 11th. Tumor hardening, and the force of the pulsation much diminished; appetite pretty good; general health much improved.

From this date, the case progressed favorably. The tumor diminished in size from day to day, although the pressure was not kept up longer than eight hours out of the twenty-four; and upon some days the patient could not be prevailed upon to wear the instrument at all.

On the 29th, the limb measured, around the knee, fifteen and a half inches; but still a perceptible thrill within the tumor. The patient being unwilling to remain longer in bed, an elastic knee-cap was applied, and, with the assistance of a crutch, he moved about the house. At the end of a week, spent in this manner, although his general condition was much improved, there was a manifest increase in the force of the pulsation within the tumor, particularly upon the cardiac side.

Upon the 7th of April, I prevailed upon him to take to his bed again, and permit a reapplication of the pressure. The instrument was then worn, steadily, for seventy-two hours; at the end of which time, upon removing it, I had the satisfaction of finding that the pulsation had entirely ceased; since then it has not reappeared. The limb, at this time, measured in circumference but two inches more than the other.

In two weeks from the last date, he was able, with the assistance of a crutch and walking-stick, to walk to his place of business, a distance of about a quarter of a mile. His employment was such that he could sit most of the time with the limb elevated. Some œdema of the foot and ankle usually took place in the after-part of the day, which, however, generally subsided at night. Complained of numbness on the dorsum and outer side of the foot, which continued to trouble him, more or less, during the greater part of the following summer. I saw him occasionally afterwards. The limb gradually recovered strength, as the circulation became more perfect.

*December* 12*th*, 1854. Saw my patient this morning, and made a careful examination of the limb. With the exception of a little thickening of the tissues within the popliteal space, not the slightest trace of the original tumor can be detected. He is, so far as the aneurism is concerned, quite well.

## Figures and Tables

**Figure f1:**